# Securin Enhances the Anti-Cancer Effects of 6-Methoxy-3-(3′,4′,5′-Trimethoxy-Benzoyl)-1H-Indole (BPR0L075) in Human Colorectal Cancer Cells

**DOI:** 10.1371/journal.pone.0036006

**Published:** 2012-04-26

**Authors:** Ho-Hsing Tseng, Qiu-Yu Chuah, Pei-Ming Yang, Chiung-Tong Chen, Jung-Chi Chao, Ming-Der Lin, Shu-Jun Chiu

**Affiliations:** 1 Department of Life Science, Tzu Chi University, Hualien, Taiwan R.O.C.; 2 Department of Pharmacology, College of Medicine, National Taiwan University, Taipei, Taiwan R.O.C.; 3 Institute of Biotechnology and Pharmaceutical Research, National Health Research Institutes, Zhunan, Taiwan R.O.C.; 4 Department of Molecular Biology and Human Genetic, Tzu Chi University, Hualien, Taiwan R.O.C.; Institute of Pathology, Germany

## Abstract

BPR0L075 [6-methoxy-3-(3′,4′,5′-trimethoxy-benzoyl)-1H-indole] is a novel anti-microtubule drug with anti-tumor and anti-angiogenic activities *in vitro* and *in vivo*. Securin is required for genome stability, and is expressed abundantly in most cancer cells, promoting cell proliferation and tumorigenesis. In this study, we found that BPR0L075 efficiently induced cell death of HCT116 human colorectal cancer cells that have higher expression levels of securin. The cytotoxicity of BPR0L075 was attenuated in isogenic securin-null HCT116 cells. BPR0L075 induced DNA damage response, G_2_/M arrest, and activation of the spindle assembly checkpoint in HCT116 cells. Interestingly, BPR0L075 induced phosphorylation of securin. BPR0L075 withdrawal resulted in degradation of securin, mitotic exit, and mitotic catastrophe, which were attenuated in securin-null cells. Inhibition of cdc2 decreased securin phosphorylation, G_2_/M arrest and cell death induced by BPR0L075. Moreover, BPR0L075 caused cell death through a caspase-independent mechanism and activation of JNK and p38 MAPK pathways. These findings provided evidence for the first time that BPR0L075 treatment is beneficial for the treatment of human colorectal tumors with higher levels of securin. Thus, we suggest that the expression levels of securin may be a predictive factor for application in anti-cancer therapy with BPR0L075 in human cancer cells.

## Introduction

Microtubules are a component of the cytoskeleton and are composed of α-tubulin and β-tubulin heterdimers [1]. Microtubules are especially important in mitosis and cell division, and this has meant that microtubules have become a target for anticancer drugs [2], [3]. According to different tubulin-binding sites, anti-microtubule drugs are classified into three classes: the paclitaxel site, the vinca alkaloid site, and the colchicines domain. Anti-microtubule drugs could induce either microtubule stabilization, such as seen with paclitaxel, or destabilization, as seen with vinblastine and colchicine [4]. BPR0L075 [6-methoxy-3-(3′,4′,5′-trimethoxy-benzoyl)-1H- indole], a Combretastatin A-4 (CA-4) analog, which is derived from the South African tree *Combretum caffrum*, is a novel synthetic anti-microtubule drug that inhibits tubulin polymerization by binding to the colchicine domain [5], and was approved by the U.S. FDA for phase 1 clinical trials in 2010. Recent studies have reported that BPR0L075 exerts not only anti-mitotic and anti-tumor activities but also anti-angiogenic activity *in vitro* and *in vivo* [6], [7].

Mitotic catastrophe is a form of cell death that results from abnormal mitosis [8]. It can be triggered by drugs influencing microtubule stability, various anticancer drugs, ionizing radiation and mitotic failure induced by cell cycle checkpoint defect [8], [9]. Furthermore, mitotic catastrophe can be caspase-dependent or –independent [10]. Biochemical features of mitotic catastrophe are prolonged spindle assembly checkpoint (SAC) signaling, aberrant levels of cyclin B1, and signaling via Cdk1 [11]. Cell death caused by mitotic catastrophe might occur during or after mitosis [12]. Anti-microtubule drugs, such as docetaxel and Combretastatin-A4 prodrug (CA4P), induce cancer cell death through mitotic catastrophe [13], [14]. These findings suggest a potential role of BPR0L075 in inducing mitotic catastrophe in human cancer cells. Whether or not mitotic catastrophe induced by BPR0L075 treatment contributes to cancer cell death is worthy of investigation.

Securin, also known as the pituitary tumor transforming gene (PTTG1), was first isolated from rat pituitary tumor cells [15]. Securin is a multi-functional protein that regulates sister chromatin segregation in mitosis [16], DNA repair [17], gene transcription [18], metabolism and organ development [19]–[21]. In normal tissue, securin expression is high in the testis and low in the thymus, colon and small intestine [22]. In contrast, securin has been identified as an oncogene and a marker of invasiveness due to its overexpression in a variety of tumors [23], and has been found to regulate tumor cell proliferation and tumorigenesis [24], [25]. In our previous studies, anticancer agents such as oxaliplatin and fisetin were shown to induce cancer cell death through down-regulation of securin expression [26]–[28]. BPR0L075 possesses anti-tumor and anti-angiogeneic activities by inhibiting the function of microtubules, especially at the metaphase to anaphase transition in mitosis [6], [7]. Securin is also a protein that is involved in control of the metaphase-anaphase transition and anaphase onset. However, the effectiveness of BPR0L075 in patients with cancers highly-expressing securin is still unknown.

In this study, we investigated the role of securin in interfering with sensitivity to BPR0L075 in human cancer cells and showed for the first time that phosphorylation and destabilization of securin enhances sensitivity to the microtubule de-stabilizing compound BPR0L075 in HCT116 human colorectal cancer cells. We further elucidated the molecular mechanisms of the cell death induced by BPR0L075 in HCT116 cells. Our findings indicate that securin appears to be a suitable clinical target for BPR0L075 treatment.

## Results

### The anticancer effects of BPR0L075 were correlated with the expression levels of securin in human cancer cells

**Figure 1:**
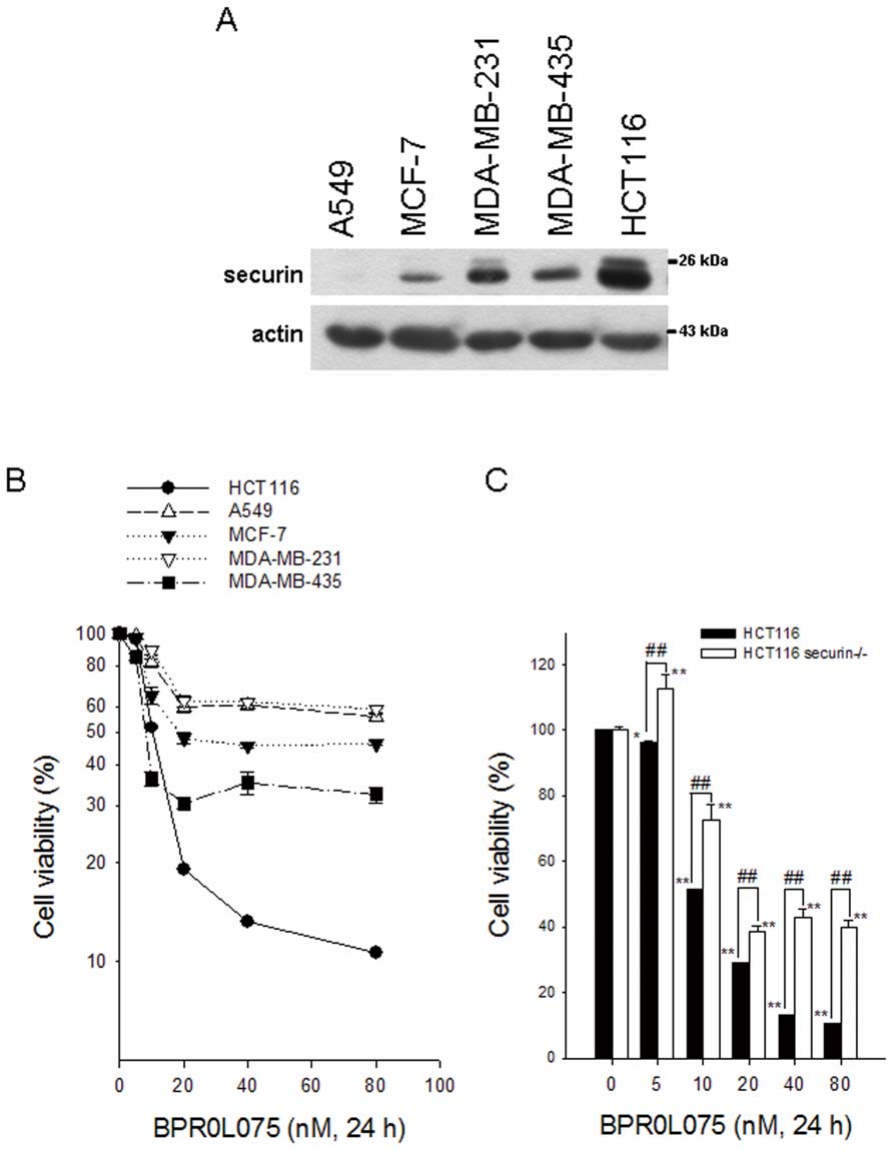
BPR0L075 induced cytotoxicity in various human cancer cell lines with different expression levels of endogenous securin. (A) The levels of securin in A549, MCF-7, MDA-MB-231, MDA-MB-435 and HCT116 cells were characterized by Western blot analysis. (B) Cells were treated with the indicated concentrations of BPR0L075 for 24 h. The cell viability was examined by MTT assay. (C) The cell viability of securin-wild-type and -null HCT116 cells was measured by MTT assay. p<0.01(**) indicates a significant difference between BPR0L075-treated and untreated samples. p<0.01(##) indicates a significant difference between securin-wild-type and -null HCT116 cells.

To investigate the role of securin in the anticancer effects of BPR0L075, several human cancer cells with different securin expression levels, including lung cancer cells (A549), human breast cells (MCF-7 and MDA-MB-231), melanoma (MDA-MB-435) and colorectal cancer cells (HCT116), were used (Fig. 1A). After treatment with BPR0L075 for 24 h, their cell viability was efficiently inhibited. Among them, HCT116 cells with the highest expression level of securin exhibited the greatest sensitivity to BPR0L075 (Fig. 1B). To ascertain the role of securin in the cytotoxicity of BPR0L075, HCT116 cells were treated with BPR0L075 and the cell viability was then examined by MTT assay. As expected, the cytotoxicity of BPR0L075 was reduced in securin-null HCT116 cells (Fig. 1C), indicating that securin expression enhanced the anticancer effects of BPR0L075.

### Loss of securin expression attenuated the cytotoxicity of BPR0L075 through decreases of G_2_/M arrest and apoptosis in HCT116 colorectal cancer cells

**Figure 2:**
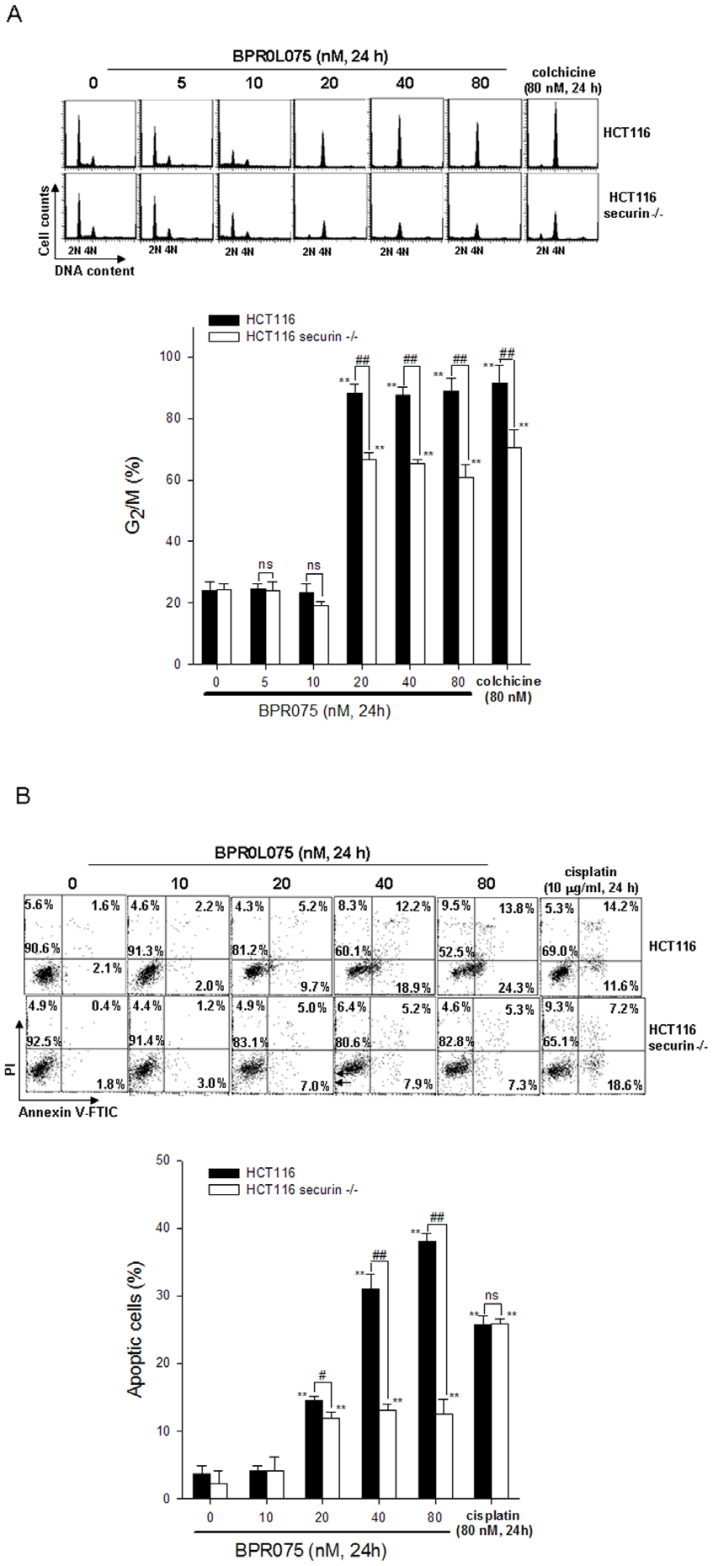
Effects of BPR0L075 on cell cycle progression and apoptosis in securin-wild-type and -null HCT116 cells. Cells were treated with 5 to 80 nM BPR0L075 for 24 h. (A) The cell cycle distribution was analyzed by flow cytometry. The percentages of G_2_/M cells were quantified. (B) Cell apoptosis was determined by Annexin-V/PI double staining. Apoptotic cells were quantified by both Annexin-V positive and Annexin-V/PI double positive cells. p<0.01(**) indicates a significant difference compared to untreated samples. p<0.01(##) indicates a significant difference between securin-wild-type and -null HCT116 cells.

To further characterize the role of securin in BPR0L075-induced cytotoxicity, cell cycle progression and cell death were analyzed by flow cytometry. BPR0L075 was found to induce G_2_/M arrest (Fig. 2A) and apoptosis (Fig. 2B) in HCT116 cells, which was attenuated in securin-null cells. Interestingly, colchicine-induced G_2_/M arrest was also reduced in securin-null HCT116 cells (Fig. 2A), whereas cisplatin induced similar fractions of apoptosis in securin-wild-type and -null HCT116 cells (Fig. 2B). These results suggested that securin is specifically required for the anticancer effects of microtubule-targeting drugs.

### Securin enhanced BPR0L075-induced DNA damage response and spindle assembly checkpoint in HCT116 cells

**Figure 3:**
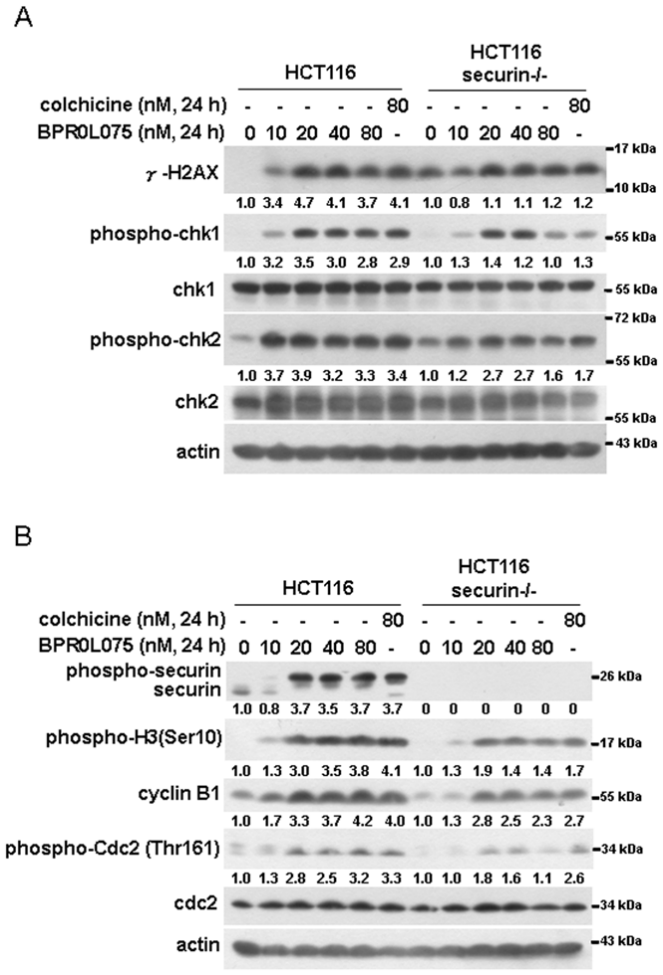
Comparison of the effects of BPR0L075 on DNA damage response and spindle assembly checkpoint activation in securin-wild-type and -null HCT116 cells. Cells were treated with 10∼80 nM BPR0L075 for 24 h. (A) The levels of γ-H2AX, phospho-chk1, phospho-chk2, total chk1 and chk2 were analyzed by Western blot in BPR0L075-treated securin-wild-type and -null HCT116 cells. (B) The levels of securin, phospho-H3 (Ser10), cyclin B1, phospho-cdc2 (Thr161) and total cdc2 were ascertained by Western blot analysis.

Anticancer agents can cause DNA damage of cancer cells and consequently activate cell cycle checkpoints, leading to cell cycle arrest, and allow the cells to repair before entering mitosis. If the cells fail to repair, cell death through apoptosis will ensue [29], [30]. γ-H2AX is regarded as a checkpoint maintenance factor, and its dephosphorylation enables resumption of the cell cycle after DNA damage is repaired [31]. To investigate whether BPR0L075 induced a DNA damage response in HCT116 cells, the protein levels of γ-H2AX after BPR0L075 treatment were analyzed using western blot. BPR0L075 induced γ-H2AX expression in both securin-wild-type and -null HCT116 cells (Fig. 3A). In response to DNA damage, two distinct kinase signaling cascades, the ATM/Chk2 and ATR/Chk1 pathways, are activated [32]. The levels of phosphorylation of Chk1 and Chk2 were elevated by BPR0L075 treatment in both securin-wild-type and -null HCT116 cells. Moreover, the activation of Chk1 and Chk2 was higher in the securin-wild-type cells than in the securin-null HCT116 cells after BPR0L075 treatment (Fig. 3A).

Treatment of cells with microtubule inhibitors results in activation of the mitotic spindle assembly checkpoint (SAC), leading to mitotic arrest before anaphase [33]. Besides, activation of SAC also inhibits the anaphase-promoting complex/cyclosome (APC/C), leading to the stabilization of securin and cyclin B1 [34]. To elucidate whether BPR0L075 activated SAC, HCT116 cells were treated with BPR0L075 and the expression levels of SAC-related mitotic markers such as phospho-Histone H3 (Ser10), cyclin B1, and phospho-cdc2 (Thr161) were examined. These mitotic markers were induced in both securin-wild-type and -null HCT116 cells (Fig. 3B). However, the extent of SAC activation was lower in securin-null cells (Fig. 3B). In addition, the expression of phospho-securin was induced by more than 10 nM BPR0L075 in wild-type HCT116 cells (Fig. 3B), which was correlated with the increase of G_2_/M arrest (Fig. 2A). Securin is enriched in G2/M phase and degraded after mitotic exit [35]. Consistently, the securin expression was reduced by 10 nM BPR0L075, in which G2/M arrest was not occurred (Fig. 2A and 3B). Therefore, BPR0L075 induced DNA damage and SAC, which can be enhanced in the presence of securin in HCT116 cells.

### BPR0L075 induced securin phosphorylation and affected the stability of mitotic regulatory molecules in HCT116 cells

**Figure 4:**
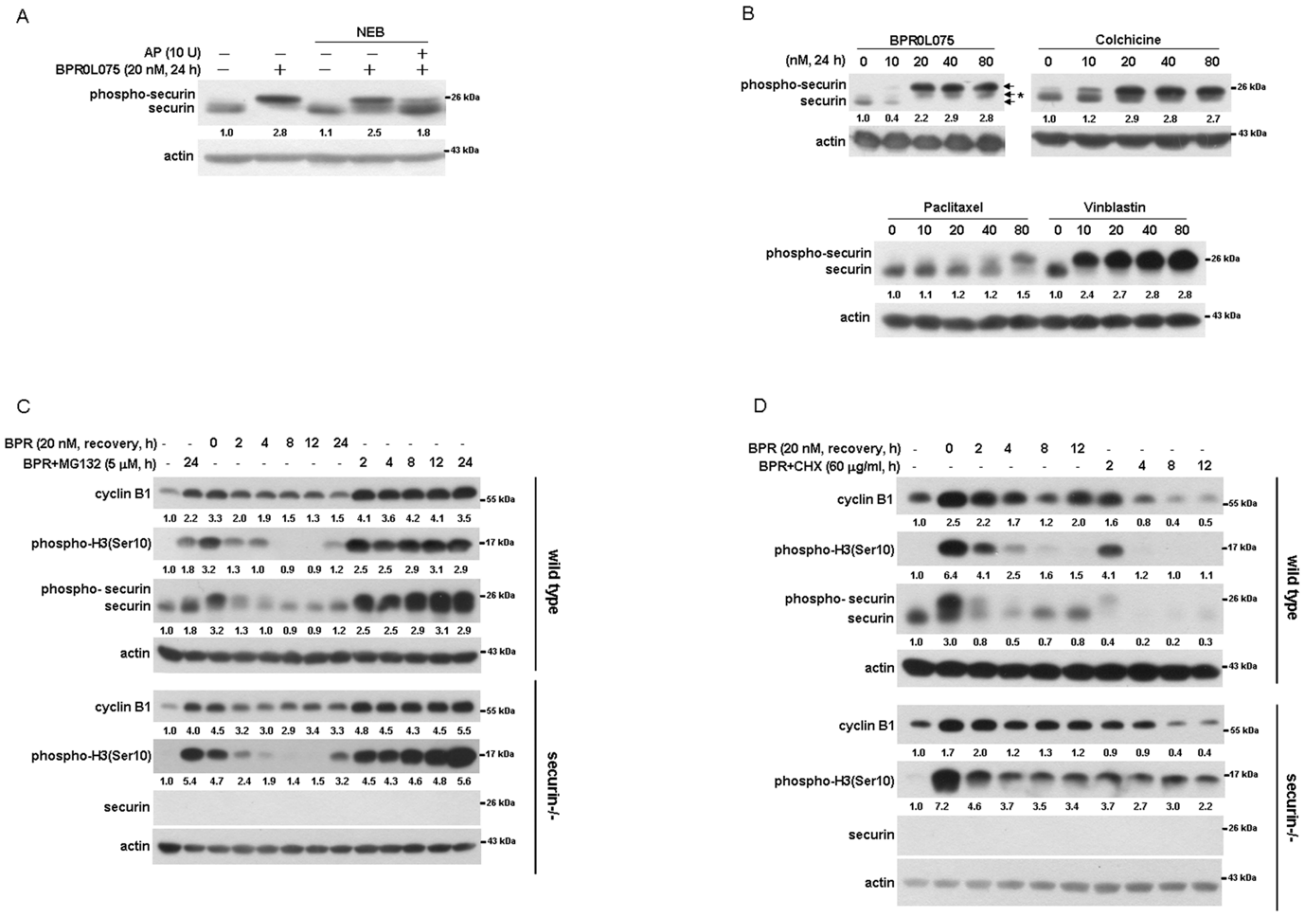
Effects of BPR0L075 on phosphorylation and instability of securin in HCT116 cells. (A) Cells were treated with 20 nM BPR0L075 for 24 h. Cell lysates were subjected to alkaline phosphatase assay and the levels of securin were determined by Western blot. (B) Cells were treated with 0 to 80 nM BPR0L075, colchicine, paclitaxel and vinblastine for 24 h. (C and D) Cells were treated with or without MG132/cycloheximide for 2 to 24 h after 24 h of BPR0L075 treatment. The cell lysates were subjected to Western blot analysis using antibodies specific for cyclin B1, phospho-H3 (Ser10) and securin.

It was noted that securin tended to be significantly band-shifted after BPR0L075 treatment in HCT116 cells (Fig. 3B). To explore whether the band-shift of securin was a result of its phosphorylation, BPR0L075-treated protein extracts were incubated with alkaline phosphatase (AP) and then analyzed by western blot. The band-shift of securin was decreased by AP (Fig. 4A), suggesting that BPR0L075 induced securin phosphorylation. To further confirm this phenomenon, several anti-microtubule drugs, including colchicine, paclitaxel, and vinblastine, were used to induce mitotic arrest. As shown in Fig. 4B, treatment with anti-microtubule drugs also resulted in band-shift of securin. Intriguingly, an intermediate migrating band (as indicated by asterisk) of securin was observed in BPR0L075-treated cells (Fig. 4B). Since six phosphorylation sites on securin have been identified [36], this band might represent a transient hypophosphorylated form of securin. Indeed, an *in vitro* securin phosphorylation assay shows two slower migration bands of phosphorylated securin [37].

The stability of securin depends on its phosphorylation state. Hyperphosphorylated forms are rapidly destroyed via the Skp1/Cul1/F-box protein complex (SCF) E3 ubiquitin ligase [38]. To examine whether BPR0L075-induced securin phosphorylation affected its protein stability, cells were treated with 20 nM BPR0L075 for 12 h and then recovered in medium containing either the proteasome inhibitor MG132 or the protein synthesis inhibitor cycloheximide (CHX) for 2–24 h. It was found that, after BPR0L075 withdrawal, the phosphorylated form of securin was rapidly degraded, and addition of MG132 blocked its degradation (Fig. 4C). In contrast, the hypophosphorylated form of securin was increased during cell recovery, which could be blocked by CHX treatment (Fig. 4D), suggesting that securin is re-synthesized after recovery from BPR0L075. The degradation rate of securin was similar to that of other mitotic regulatory molecules including cyclin B1 and phospho-histone H3 in wild-type HCT116 cells (Fig. 4C and 4D). Interestingly, the accumulation of phospho-histone H3 was higher in MG132-treated securin-null cells (Fig. 4C), and the decreases of cyclin B1 and phospho-histone H3 were lower in CHX-treated securin-null cells (Fig. 4D). These results showed that BPR0L075 treatment induced instability of mitotic regulatory molecules in the presence of securin.

### BPR0L075 induced mitotic catastrophe in HCT116 cells

**Figure 5:**
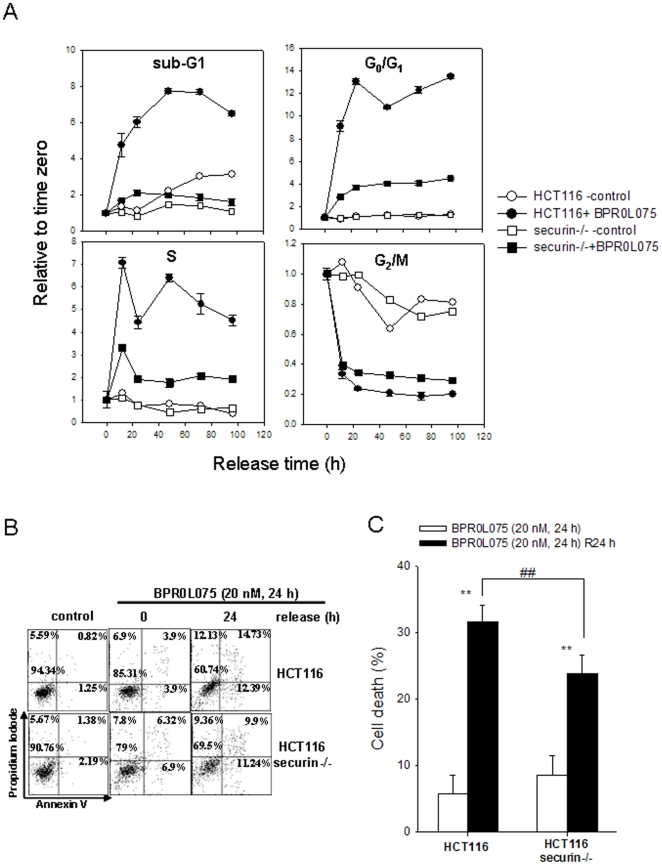
Effects of BPR0L075 withdrawal on cell cycle progression and apoptosis in securin-wild-type and -null HCT116 cells. (A) Cells were treated with 20 nM BPR0L075 for 12 h and BPR0L075 withdrawal for 12 to 96 h. The cell cycle distribution was determined by flow cytometry. (B and C) Cells were treated with BPR0L075 for 24 h and BPR0L075 withdrawal or no withdrawal for 24 h. The percentage of dead cells (Annexin positive and Annexin/PI double positive) were determined by Annexin-V/PI staining. p<0.01(**) indicates a significant difference between BPR0L075-treated and untreated samples. p<0.01(##) indicates a significant difference between securin-wild-type and -null HCT116 cells.

Mitotic catastrophe is a form of cell death during or after abnormal mitosis [8]. Our results suggested that BPR0L075 induced phosphorylation of securin, which may destabilize mitotic regulatory molecules and consequently promote mitotic catastrophe in HCT116 cells. To address this possibility, securin-wild-type and -null HCT116 cells treated with 20 nM BPR0L075 for 12 h were recovered in culture medium for 12–96 h, and cell cycle progression and apoptosis were then analyzed using flow cytometry. The results indicated that, after BPR0L075 removal, the G_2_/M fraction was decreased and cell cycle progression was resumed in securin-wild-type and -null HCT116 cells (Fig. 5A). However, the decreases of the G_2_/M fraction in securin-wild-type cells were more significant than those in the securin-null cells, which was reflected by the increases in G_0_/G_1_ and S phase cells in wild-type cells (Fig. 5A). In addition, the increases in the sub-G_1_ fraction were also higher in securin-wild-type cells (Fig. 5A), suggesting that securin expression promoted mitotic catastrophe in HCT116 cells. Furthermore, cell apoptosis after BPR0L075 withdrawal was analyzed by annexin V/PI double staining. Consistently, more cell apoptosis in securin-wild-type cells was induced after cell recovery for 24 h (Fig. 5B and 5C).

### BPR0L075 induced phosphorylation of securin, G_2_/M arrest and cytotoxicity through a cdc2 (cdk1)-dependent pathway

**Figure 6:**
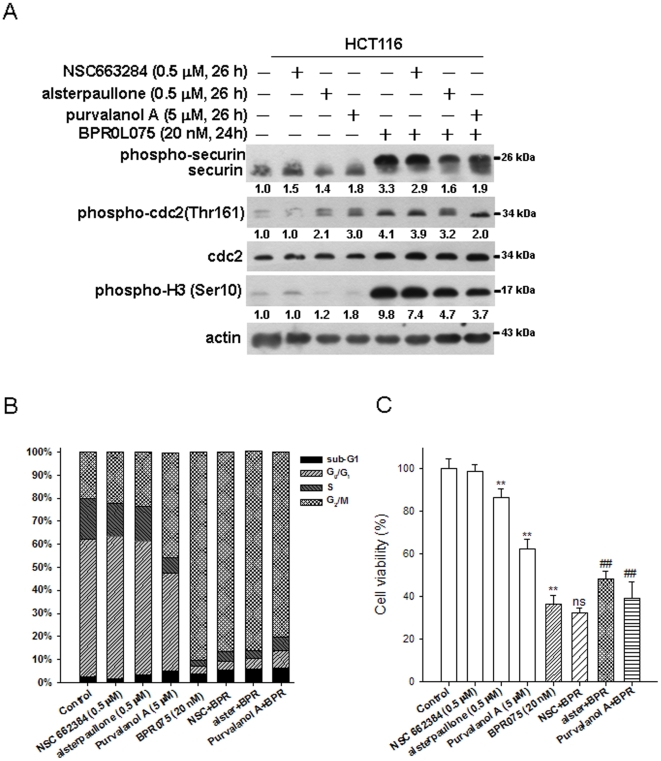
Effects of inhibitors of cdc2/cdk and cdc25 on BPR0L075-induced phosphorylation of securin, cell cycle progression and cytotoxicity in HCT116 cells. Cells were pretreated with NSC663284, alsterpaullone and purvalanol for 2 h prior to exposure to 20 nM BPR0L075 for 24 h. (A) The levels of securin, phospho-cdc2 (Thr161), phospho-H3 (Ser10) and total cdc2 were analyzed by Western blot. (B) The cell cycle distribution was determined by flow cytometry. (C) The cell viability was analyzed by MTT assay. p<0.01(**) indicates a significant difference between alsterpaullone (0.5 µM), Purvalanol (0.5 µM) and BPR075 (20 nM) alone in comparison with control. p<0.01(##) indicates a significant difference between BPR0L075 alone and pre-treatment with alsterpaullone and purvalanol.

Securin is phosphorylated by cdc2 (cdk1) [39]. To investigate whether cdc2 signaling is responsible for the BPR0L075-induced phosphorylation of securin, the effects of cdc2, CDK and cdc25 specific inhibitors (alsterpaullone, purvalanol or NSC 663284, respectively) on BPR0L075-induced phosphorylation of securin were monitored. The phosphorylation of securin was partially decreased by cdc2/CDK inhibitors (Fig. 6A). In addition, we also showed that inhibition of cdc2 or CDK reduced BPR0L075-induced G_2_/M arrest and cytotoxicity in securin-wild-type HCT116 cells (Fig. 6B and 6C). These results suggest that in response to BPR0L075 treatment, cdc2 phosphorylated securin, leading to higher G_2_/M arrest and thus facilitating the cytotoxicity of BPR0L075 in HCT116 cells.

### BPR0L075-induced cell death through activation of the JNK and p38 MAPK pathways and a caspase-independent mechanism in HCT116 cells

**Figure 7:**
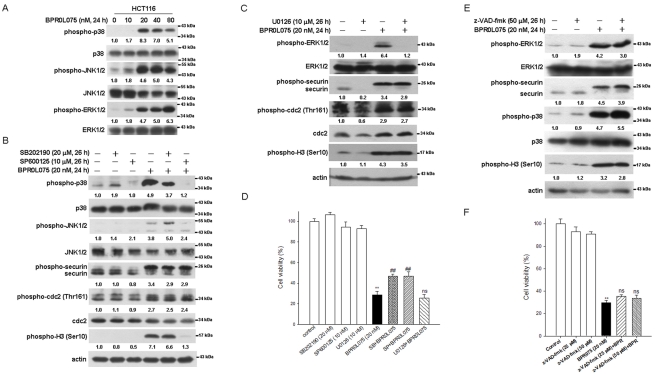
Effects of MAPK kinases on BPR0L075-induced phosphorylation of securin and cytotoxicity in HCT116 cells. (A) Cells were treated with 0 to 80 nM BPR0L075 for 24 h. The levels of phospho-p38, -JNK1/2, and -ERK1/2, and total p38, JNK1/2 and ERK1/2 were determined by Western blot. (B and C) Cells were pretreated with SB202190, SP600125 and U0126 for 2 h prior to exposure to 20 nM BPR0L075 for 24 h. The levels of phospho-p38, -JNK1/2, -ERK1/2, -cdc2 (Thr161), and -H3 (Ser10), and total p38, JNK1/2, ERK1/2 and cdc2 were analyzed by Western blot. (D) The cell viability was determined by MTT assay. (E and F) Cells were pretreated with z-VAD-fmk for 2 h then exposed to 20 nM BPR0L075 for 24 h. The levels of phospho-p38, -ERK1/2, and -H3 (Ser10), and total p38, ERK1/2 and securin were analyzed by Western blot. The cell viability was analyzed by MTT assay. p<0.01(**) indicates a significant difference between BPR0L075-treated and untreated samples. p<0.01(##) indicates a significant difference between BPR0L075 alone and pre-treatment with SB202190 and SP600125.

In response to external stresses or damage, cells usually activate the JNK or p38 MAPK pathways, leading to cell death [40], or the ERK pathway for survival [41]. It has been reported that activation of p38 MAPK or inhibition of ERK is involved in the apoptosis induced by the anti-microtubule drug nocodazole alone or combination with paclitaxel [42], [43]. To address the role of MAPK pathways in BPR0L075-induced cell death in securin-wild-type HCT116 cells, the activations of p38 MAPK, JNK and ERK were analyzed by western blot. The p38 MAPK, JNK and ERK pathways were activated by BPR0L075 (Fig. 7A). Specific inhibitors of p38 MAPK, JNK and ERK (SB2021900, SP600125 and U0126, respectively) blocked the BPR0L075-induced activation (Fig. 7B and 7C). However, inhibition of the p38 MAPK, JNK and ERK pathways did not affect BPR0L075-induced phosphorylation of securin (Fig. 7B and 7C). In addition, only SP600125 inhibited BPR0L075-induced phospho-Histone H3 (Fig. 7B).

We further analyzed the effects of MAPK inhibitors on BPR0L075-induced cytotoxicity by MTT assay. Treatment with SB202190, SP600125 or U0126 alone did not affect cell survival in HCT116 cells (Fig. 7D). Moreover, SB202190 and SP600125 were able to rescue cells from BPR0L075-induced cytotoxicity (Fig. 7D). These results indicate that activation of the p38 MAPK and JNK pathways are required for BPR0L075-induced cell death in HCT116 cells. To further investigate whether caspase activation is involved in BPR0L075-induced cell death, the cell viability of cells pretreated with pan caspase inhibitor, z-VAD-fmk, combined with BPR0L075 treatment was analyzed by MTT assay. We found that inhibition of caspase did not affect the cell viability after BPR0L075 treatment (Fig. 7E). Moreover, BPR0L075-induced activation of the p38 MAPK and ERK pathways, as well as phosphorylation of securin and Histone H3, were not affected. These results imply that BPR0L075 induced cell death through a caspase-independent pathway in HCT116 cells.

## Discussion

Securin, an oncogene, is overexpressed in various cancer cells and is responsible for promoting cell proliferation and tumorigenesis [24], [25]. Previously, it has been reported that securin levels are down-regulated in HCT116 cell death induced by chemotherapeutic drugs [26], [44], suggesting that it might be a target for cancer therapy. In this study, we found that the cytotoxicity of the anti-microtubule drug BPR0L075 was positively correlated with the expression levels of securin in various cancer cells. Securin was phosphorylated by treatment with BPR0L075 as well as other anti-microtubule drugs including colchicines, paclitaxel, and vinblastine, in HCT116 cells. The accumulation of phosphorylated securin was accompanied by higher G2/M arrest and cytotoxicity. The phosphorylation of securin further leads to its destabilization and then promotes mitotic exit and mitotic catastrophe of HCT116 cells. Therefore, we propose that securin is an important target for the anticancer effects of BPR0L075 in human colorectal cancer cells.

Mitotic catastrophe could be caused by drugs that target microtubules or affect the progression of mitosis [8], [9]. Many different cell fates could be caused by mitotic catastrophe. First, cells can die without mitotic exit. Second, after mitotic exit and reaching of the G1 phase, cells undergo cell death or senescence [12]. It has been reported that docetaxel, a microtubule-stabilizing agent, induces cell death through mitotic catastrophe in human breast cancer cells [14]. Furthermore, another microtubule-stabilizing agent, combretastatin-A4 prodrug (CA4P), induces mitotic catastrophe after mitotic arrest in chronic lymphocytic leukemia cells [13]. Our results show that BPR0L075 induced more extensive cell death after drug withdrawal in securin-wild-type HCT116 cells than in securin-null cells. In addition, recent study showed that prolonged mitotic arrest induced by anti-microtubule drugs could lead to more cell death [45]. Moreover, we found that BPR0L075 treatment for 12 to 24 h induced more cell death after withdrawing BPR0L075 as compared with treatment for 4 or 8 h (data not shown). These findings suggest that BPR0L075 induced mitotic catastrophe through induction of prolonged mitotic arrest in human colorectal cancer cells.

Nocodazole treatment induces mitotic arrest and phosphorylation of securin in HCT116 and U2OS cells [46]. Consistently, in this study we found that BPR0L075 and other anti-microtubule drugs induced securin phosphorylation in HCT116 cells. It has been reported that securin is phosphorylated by cdc2 (cdk1) [39]. MAPK kinase also induces securin phosphorylation and regulates its transactivation function [47]. In response to DNA damage, securin is phosphorylated by DNA-PK (DNA-dependent protein kinase), which contributes to the blocking of sister chromatin separation [17], [48]. As shown by our results, BPR0L075-induced phosphorylation of securin was only partially inhibited by cdc2/CDK specific inhibitor. These results demonstrated that BPR0L075 induced securin phosphorylation partly through cdc2 activation. Therefore, there might be other kinases that induce phosphorylation of securin. However, BPR0L075-induced activation of MAPK kinases was not involved in phosphorylation of securin. Therefore, we propose that BPR0L075 induces cell death through the p38 MAPK and JNK pathways, which is independent of phosphorylation of securin.

Securin has been reported to interact with the DNA repair protein Ku70. When cells suffer DNA damage, DNA-PK phosphorylates securin to trigger cell cycle arrest. Then, the interaction of securin and Ku70 is repressed, thus releasing Ku70 to promote DNA repair [17], [48]. In contrast, securin overexpression may delay mitosis progression and sister chromatid separation during DNA damage through the inhibition of Ku70 [48]. In our study, BPR0L075 induced higher G_2_/M arrest in securin-wild-type HCT116 cells than in securin-null cells, suggesting that a greater repair ability and elevated checkpoint activation were elicited in the presence of securin, which may result from the phosphorylation and destabilization of securin by BPR0L075.

**Figure 8:**
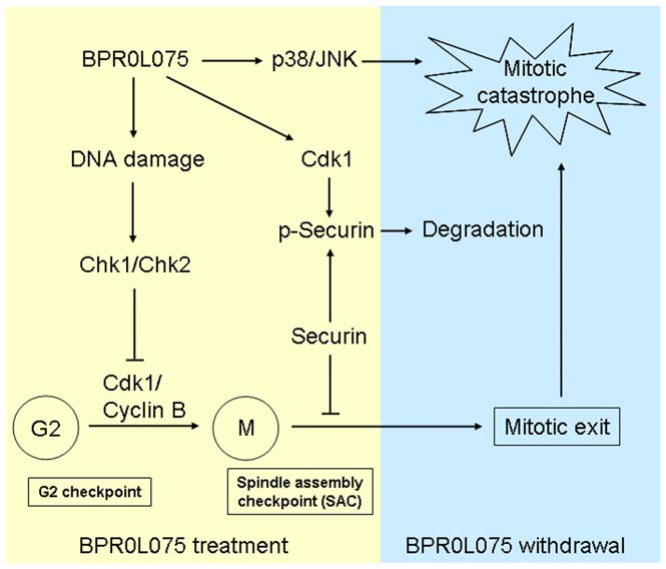
Proposed model of the anti-cancer effects induced by BPR0L075 in HCT116 cells. BPR0L075 induced DNA damage, leading to Chk1/Chk2 activation, inhibition of Cdk1 activity and G2 arrest. It also modulated Cdk1-dependent phosphorylation of mitotic regulatory molecules to activate the spindle assembly checkpoint, which was attenuated in the presence of securin. After BPR0L075 withdrawal, cells of the G_2_/M fraction underwent mitotic catastrophe, which was enhanced by phosphorylation and destabilization of securin. Moreover, BPR0L075 caused cell death through a caspase-independent mechanism and activation of JNK and p38 MAPK pathways.

Expression of securin has been found in various cancers to be correlated with a poor clinical outcome [49], [50]. In this study, we found that BPR0L075 induced DNA damage and modulated mitotic regulatory molecules to activate the spindle assembly checkpoint in HCT116 cells. After BPR0L075 withdrawal, cells of the G_2_/M fraction underwent mitotic catastrophe, which was enhanced by phosphorylation and destabilization of securin. Moreover, BPR0L075 caused cell death through a caspase-independent mechanism and activation of JNK and p38 MAPK pathways (Fig. 8). These findings provided evidence for the first time that BPR0L075 treatment is beneficial for the treatment of human colorectal tumors with higher levels of securin. As BPR0L075 was approved by the U.S. FDA for phase 1 clinical trial in 2010, the expression levels of securin could be a predictive factor for deciding the priority of anti-cancer therapy using BPR0L075 in human cancer cells. Our results also indicate that targeting securin may be a suitable strategy to broaden the clinical use of BPR0L075 for various cancers overexpressing securin.

## Materials and Methods

### Materials

The compound BPR0L075 was synthesized at the Division of Biotechnology and Pharmaceutical Research, National Health Research Institutes, Zhuman, Taiwan, ROC. BPR0L075, a white solid, was obtained in a 72% yield from 6-methoxyindole and 3,4,5-trimethoxybenzoyl chloride and was dissolved in dimethyl sulfoxide. Propidium iodide (PI; p4170), 3-(4,5-Dimethylthiazol-2yl)-2,5- diphenyltetrazolium bromide (MTT; M5655), colchicines (C9754) and purvalanol (P4484) were purchased from Sigma Chemical Co. Antibodies specific to ERK(C14), p38α (C-20) and JNK(C-17) were purchased from Santa Cruz Biotechnology. Anti-phospho-Histone H2A.X (Ser139) (#05-636) and phospho-Histone H3 (Ser10) (#05-806) antibodies were purchased from Upstate. Anti-securin (ab-3305) antibody was purchased from Abcam. Anti-cdc2 (Ab-1) antibody was purchased from Oncogene Sciences Products. Anti-actin (MAB1501) antibody was purchased from Chemicon International. Phospho-cdc2 (Thr161) (#9114), phospho-chk1 (Ser345) (#2341), phospho-chk2 (Thr68) (#2661), phospho-SAPK/JNK (Thr183/Tyr185) (#9251), phospho-ERK1/2 (Thr202/Tyr204) (#9106), phospho-p38 MAP kinase (Thr180/Tyr182) (#9211), chk1 (#2345), and chk2 (#2662) antibodies were purchased from Cell Signaling Technology. Cyclin B1 (Ab-2) and p21 (Ab-1) antibodies, NSC663284 (#217692), alsterpaullone (#126870), MG132 (#474790), cycloheximide (CHX; #2379763), SB202190 (#559388), SP600125 (#420119) and U0126 (#662005) were purchased from Calbiochem.

### Cell culture

Securin-wild-type and securin-null human HCT116 colorectal cancer cell lines [27], MCF-7 and MDA-MB-231 human breast cancer cell lines [51], and MDA-MD-435 human melanoma cell lines [52] were gifts from Dr. Ji-Hshiung Chen (Department of Molecular Biology and Human genetics, Tzu Chiu University, Taiwan). Securin-wild-type and securin-null human HCT116 colorectal cancer cells were routinely maintained in McCoy'5A medium (Sigma; M4892). MCF-7 and MDA-MB-231 human breast cancer cells were maintained in Dulbecco's Modified Eagle Medium (DMEM; GIBCO; 12800-058). A549 human lung cancer cell line [53] (gifts from Dr. Lih-Yuan Lin; Department of Life Science, National Tsing Hua University, Taiwan) were maintained in RPMI 1640 medium (GIBCO; 23400-021). The complete medium was supplemented with 10% fetal bovine serum (FBS). Unsynchronized cells were used in this study.

### Cell viability assay

Cell viability was determined by MTT colorimetric assay. Briefly, cells were seeded at a density of 5,000–10,000 cells per well in 96-well plates for 16–24 h. At the end of BPR0L075 treatment, the cells were washed with phosphate-buffered saline (PBS) and were re-cultured in culture medium for 2–3 days. Subsequently, the medium was replaced by new medium supplemented with 0.5 mg/ml of MTT and incubated for 4 h. The viability of the cells was determined by the measurement of formazan production converted from MTT, which can be quantified by the development of a blue–purple color in DMSO. The intensity of formazan staining was measured at 545 nm using a plate reader (OPTImax; Molecular Dynamics), and the relative percentage of viable cells was calculated by dividing the absorbance resulting from the treated cells (the average of six wells) by that of the control included in each experiment.

### Cell cycle analysis

The securin-wild-type and -null HCT116 cells were plated at a density of 500,000 cells per 35-mm culture dish and incubated for 24 h. Cells were treated with BPR0L075 at different concentrations for 24 h. After treatment, cells were trypsinized and fixed with ice-cold 70% ethanol at −20°C overnight. Fixed cells were stained with 20 µg/ml PI staining buffer (containing 1% triton X-100 and 100 µg/ml RNase A) for 30 min. Then, the samples were analyzed by flow cytometry (FACSCalibur, Becton Dickinson). DNA histograms were plotted to calculate the percentage of cells in the different cell cycle phases by ModFit LT software (Vesion 2.0, Becton Dickinson).

### Immunoblot analysis

Total cellular protein extracts were prepared according to our previous study [51]. Equivalent amounts of proteins (20–60 µg/well) were subjected to electrophoresis using 10–12% sodium dodecyl sulfate-polyacrylamide gels. After electrophoretic transfer of proteins onto polyvinlylidine fluoride membranes, the proteins were sequentially hybridized with a specific primary antibody, followed by a horseradish peroxidase-conjugated secondary antibody. The protein bands were visualized on X-ray film using the ECL detection system (Immobilon ™ Western Chemiluminescent HRP Substrate, WBKLS0500, Milipore), and a gel-digitizing software, Un-Scan-It gel (Version 5.1; Silk Scientific, Inc.), was used to quantify the relative intensity of each band on the X-ray film.

### Alkaline phosphatase assay

HCT116 cells untreated and treated with BPR0L075 were incubated for 24 h. Afterward, samples were washed with PBS, homogenized using 0.1% Triton X-100, pH 8.0 in PBS for 30 min at 4°C, and the homogenate was used for the alkaline phosphatase activity assay kit (BioVision) was used according to the manufacturer's instructions.

### Cell death detection

The cell death induced by BPR0L075 was determined by the analysis of sub-G1 phase cells and Annexin V-PI double staining assay, for which an Annexin V-PI staining kit (BioVision) was used according to the manufacturer's instructions. Cells were analyzed by flow cytometry (Becton Dickinson), and the percentages of Annexin-V-positive or PI-positive cells were calculated using ModFit LT software (Ver. 2.0, Becton-Dickinson).

### Statistical analysis

All of the data are represented as the mean ± standard error of the mean (SEM) from at least three independent experiments. Statistical comparisons were performed by one-way analysis of variance, and further *post-hoc* testing was conducted using the statistical software GraphPad Prism 4 (GraphPad Software, Inc.). A *p*<0.05 was considered statistically significant.
